# Genetic variation of γ-tocopherol methyltransferase gene contributes to elevated α-tocopherol content in soybean seeds

**DOI:** 10.1186/1471-2229-11-152

**Published:** 2011-11-07

**Authors:** Maria S Dwiyanti, Tetsuya Yamada, Masako Sato, Jun Abe, Keisuke Kitamura

**Affiliations:** 1 Laboratory of Plant Genetics and Evolution, Graduate School of Agriculture, Hokkaido University, Kita 9 Nishi 9 Sapporo 060-8589, Hokkaido, Japan

## Abstract

**Background:**

Improvement of α-tocopherol content is an important breeding aim to increase the nutritional value of crops. Several efforts have been conducted to improve the α-tocopherol content in soybean [*Glycine max *(L.) Merr.] through transgenic technology by overexpressing genes related to α-tocopherol biosynthesis or through changes to crop management practices. Varieties with high α-tocopherol content have been identified in soybean germplasms. The heritability of this trait has been characterized in a cross between high α-tocopherol variety Keszthelyi Aproszemu Sarga (KAS) and low α-tocopherol variety Ichihime. In this study, the genetic mechanism of the high α-tocopherol content trait of KAS was elucidated.

**Results:**

Through QTL analysis and fine mapping in populations from a cross between KAS and a Japanese variety Ichihime, we identified *γ-TMT3*, which encodes *γ*-tocopherol methyltransferase, as a candidate gene responsible for high α-tocopherol concentration in KAS. Several nucleotide polymorphisms including two nonsynonymous mutations were found in the coding region of *γ-TMT3 *between Ichihime and KAS, but none of which was responsible for the difference in α-tocopherol concentration. Therefore, we focused on transcriptional regulation of *γ-TMT3 *in developing seeds and leaves. An F_5 _line that was heterozygous for the region containing *γ-TMT3 *was self-pollinated. From among the progeny, plants that were homozygous at the *γ-TMT3 *locus were chosen for further evaluation. The expression level of *γ-TMT3 *was higher both in developing seeds and leaves of plants homozygous for the *γ-TMT3 *allele from KAS. The higher expression level was closely correlated with high α-tocopherol content in developing seeds. We generated transgenic Arabidopsis plants harboring GUS gene under the control of *γ-TMT3 *promoter from KAS or Ichihime. The GUS activity assay showed that the activity of *γ-TMT3 *promoter from KAS was higher than that of Ichihime.

**Conclusions:**

The genetic variation in *γ-TMT3*, which plays a major role in determining α-tocopherol concentration, provides significant information about the regulation of tocopherol biosynthesis in soybean seeds. This knowledge will help breeding programs to develop new soybean varieties with high α-tocopherol content.

## Background

The vitamin E family comprises tocopherols (α, β, γ, and δ forms) and tocotrienols (α, β, γ, and δ forms). All isoforms possess lipid antioxidant activity, and α-tocopherol possesses the highest vitamin E activity in mammals [1,2]. Vitamin E is widely used as an antioxidant in foods and oils, as a nutrient additive in poultry and cattle feeds to improve meat quality, and as a supplement in the human diet to help prevent diseases such as cancer and cardiovascular diseases. The market size is expected to grow because of the increasing interest in functional food and increasing demand for meat products. About 85% of commercial vitamin E is synthesized by chemical reaction [3]. This vitamin E usually includes the naturally occurring RRR-α-tocopherol and 7-stereoisomers as secondary products, whose biological activity is only 50%-74% of that of the natural α-tocopherol [4]. Thus, it is very important to increase natural vitamin E production in crops and vegetables [2].

**Figure 1:**
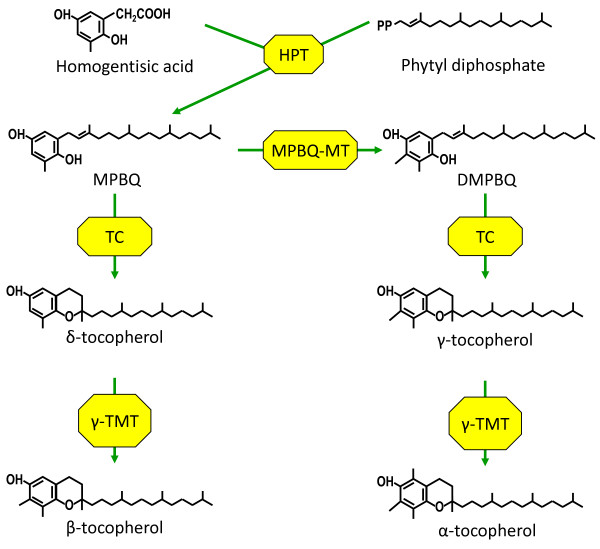
**Tocopherol biosynthetic pathway in higher plants**. Tocopherols consist of a polar chromanol ring and a lipophilic prenyl chain derived from homogentisic acid and phytyl diphosphate. The shikimate pathway produces the homogentisic acid, whereas the 2-C-methyl-d-erythritol-4-phosphate (MEP) pathway produces phytyl diphosphate. Phytyl transferase (HPT) catalyzes the reaction of phytyl diphosphate addition to homogentisic acid, producing the common precursor of the tocopherol biosynthetic pathway, methyl-6-phytyl-1,4-benzoquinone (MPBQ). MPBQ-methyltransferase (MPBQ-MT) adds a methyl alkyl to MPBQ, to produce 2,3-dimethyl-6-phytyl-plastoquinol (DMPBQ). MPBQ and DMPBQ are cyclized by tocopherol cyclase (TC) to form δ-tocopherol and γ-tocopherol, respectively. The last step of tocopherol biosynthesis is methylation of δ-tocopherol and γ-tocopherol, which produces β-tocopherol and α-tocopherol, respectively. These reactions are catalyzed by γ-tocopherol methyltransferase (γ-TMT).

Soybean (*Glycine max *(L.) Merr.) is one of the major crops for food, oil, and animal feed. In seed processing, tocopherols are extracted together with the oil fraction. The tocopherol content is only about 1.5% of the oil; nevertheless, tocopherols are critical for oxidative stability [5]. Since tocopherols contribute to both the nutritional value of seeds and the oxidative stability of soybean oil, enhancing tocopherol content in soybean will improve its market value. In common soybean cultivars, the main forms of seed tocopherols are γ-tocopherol and δ-tocopherol, which account for 60% to 70% and 20% to 25% of the total tocopherol, respectively. The proportion of α-tocopherol is usually less than 10% of total tocopherol in soybean seeds [1,6,7]. There have been some efforts to improve soybean vitamin E through genetic engineering. The Arabidopsis *VTE4 *gene encodes γ-tocopherol methyltransferase (γ-TMT), which catalyzes the last step of α-tocopherol biosynthesis (Figure 1); overexpression of *VTE4 *in soybean seeds resulted in α-tocopherol elevation to 75% of total tocopherol. When *VTE4 *was coexpressed with *VTE3*, which encodes methyl-6-phytyl-1,4-benzoquinol (MPBQ)-methyltransferase (Figure 1), α-tocopherol increased to more than 95% of total tocopherol, and vitamin E activity increased to up to five times the level in nontransgenic soybean [6]. Meanwhile, overexpression of *Perilla frutescens *γ-TMT alone increased α-tocopherol to more than 90% of total tocopherol [8]. Several studies have suggested the importance of other tocopherol forms. For example, γ-tocopherol may prevent inflammation or improve kidney function, which are distinct from its antioxidant activity [9,10]. These studies triggered us to look for natural tocopherol variants, which may have unique characteristics. Such variants may make it possible to breed soybean cultivars with a wide range of α-tocopherol (from 10% to 90% of total tocopherol), and to develop soybean cultivars tailor-made for certain purposes.

Tocopherols are present in leaves, stems, flower petals, and seeds of higher plants and green algae [1,11]. While α-tocopherol is usually the predominant form in leaves, there are diverse variations of tocopherol composition in seeds [1]. For example, in soybean, rapeseed (*Brassica napus*), and Arabidopsis (*Arabidopsis thaliana*), most of the tocopherols are γ-tocopherol or δ-tocopherol; in sunflower (*Helianthus annuus*) and safflower (*Carthamus tinctorius*) seeds, the content of α-tocopherol is more than 95% of the total tocopherol content [12,13]. Variations in α-tocopherol content (α-tocopherol weight [μg] per 100 mg seed powder) and concentration (α-tocopherol as a percentage of total tocopherol) have been reported in crops such as maize (from 0.9 to 6.5 μg 100 mg^-1^), sunflower (>95% in wild type and <10% in mutants), safflower (>85% in wild type and <15% in mutants), rapeseed (α/γ-tocopherol ratio ranged from 0.54 to 1.70) and in the model plant Arabidopsis [12-16]. Previous studies have shown that variation is also present in soybean. Three soybean varieties with α-tocopherol concentration of 20% to 30%, Keszthelyi Aproszemu Sarga (KAS), Dobrogeance, and Dobrudza 14 Pancevo, were identified through analysis of more than 1,000 cultivars and varieties from soybean germplasms collections [7]. These varieties showed higher α-tocopherol content compared to typical cultivars over two planting years, indicating that high α-tocopherol content was a stable trait [7]. QTL analysis using Chinese (Hefeng 25) and Canadian (OAC Bayfield) soybean varieties revealed four QTLs for tocopherol content in linkage groups B2, C2, D1b, and I, which correspond to chromosome 14, 6, 2, and 20, respectively. However, the causal genes involved in these QTLs are yet to be identified [17].

In our previous study, the genetic characteristics of the high α-tocopherol concentration trait were evaluated in an F_2 _population derived from a cross between KAS and a typical variety, Ichihime [18]. α-Tocopherol concentration of a typical variety is less than 10% of total tocopherol [6]. Here and in our previous study [18], α-tocopherol concentration was defined as the ratio of α-tocopherol to total tocopherol, whereas α-tocopherol content was defined as the α-tocopherol weight (μg) per 100 mg soybean seed powder. The broad-sense heritability of the high α-tocopherol concentration trait was estimated to be 0.645 [18]. Two simple sequence repeats (SSR) markers, Sat_167 and Sat_243 on linkage groupK (chromosome 9) were strongly correlated with α-tocopherol concentration [18]. The relationships between tocopherol forms were also analyzed; α-tocopherol concentration had no significant correlation with total tocopherol content, whereas γ-tocopherol and α-tocopherol concentrations showed a strong negative correlation [18].

The strong negative correlation between α-tocopherol concentration and γ-tocopherol concentration suggested that a major gene involved in the biosynthesis pathway of α-tocopherol might be responsible for the trait [18]. Tocopherols are biosynthesized from two precursors, homogentisic acid (HGA) and phytyl diphosphate. The two precursors are condensed by HGA phytyl transferase, generating MPBQ. MPBQ is methylated to become 2,3-dimethyl-6-phytyl-1,4-benzoquinol (DMPBQ). MPBQ and DMPBQ are converted by tocopherol cyclase to δ-tocopherol and γ-tocopherol, respectively. The last step of the tocopherol biosynthesis pathway is methylation of δ-tocopherol and γ-tocopherol by γ-tocopherol methyltransferase (γ-TMT), yielding β-tocopherol and α-tocopherol, respectively (Figure 1) [1].

To elucidate the genetic basis of the high α-tocopherol concentration trait in KAS, we performed QTL analysis and fine mapping for α-tocopherol concentration by using the population derived from a cross between a typical variety Ichihime and the high α-tocopherol variety KAS. The *γ-TMT3*, which has high similarity to the Arabidopsis *VTE4 *gene, was located within a QTL region of approximately 75 kb. The expression level of *γ-TMT3 *was higher in developing seeds of plants with the KAS genotype, and the expression elevation was correlated with an increase in α-tocopherol content. It is also demonstrated that the transient activity of *γ-TMT3 *promoter from KAS was higher than that of Ichihime.

## Results

### Mapping the QTL responsible for the high α-tocopherol concentration trait

KAS, a soybean variety with 20% to 30% α-tocopherol concentration, was crossed to the Japanese cultivar Ichihime (α-tocopherol concentration <10%) to obtain a segregating population consisting of 122 F_2 _plants [18]. These plants were grown in the Hokkaido University greenhouse, where F_3 _seeds of each F_2 _plant were obtained and analyzed for their tocopherol composition. A molecular linkage map was constructed using 152 SSR markers that were polymorphic between Ichihime and KAS. The linkage map covered 3401 cM of the soybean genome and consisted of 20 linkage groups that corresponded to the 20 pairs of soybean chromosomes.

Two population groups were used for QTL analysis. The first population (hereafter, "F_2 _seed population") consisted of F_2 _seeds from the Ichihime × KAS cross; in this population, tocopherol concentrations were analyzed using the half-seed method (see Materials and Methods). The second population ("F_2 _plant population") consisted of F_2 _plants whose tocopherol content and concentration were evaluated by testing the F_2:3 _seeds. Multiple QTL Mapping (MQM) analysis was performed using MapQTL5, and the QTL threshold values were determined for each trait by using a 1,000-permutation test [19].

**Figure 2:**
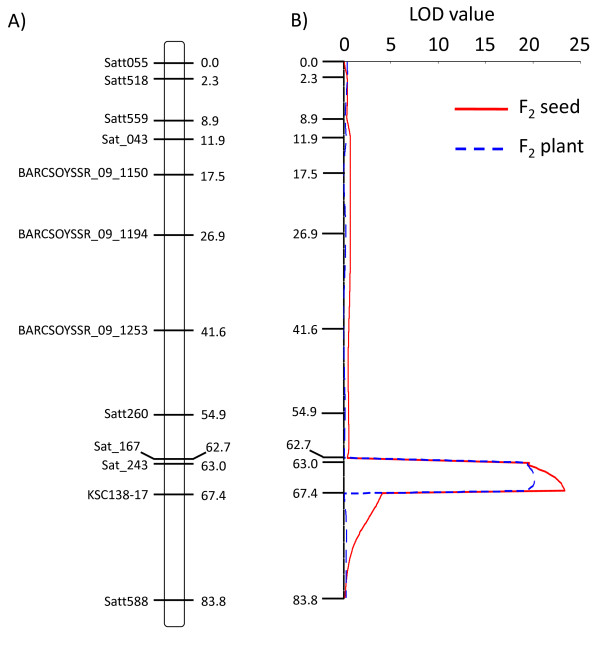
**QTL for high α-tocopherol concentration on chromosome 9**. (A). Graphical overview of the genetic map on chromosome 9. A vertical thick bar indicates soybean chromosome 9. Molecular markers and genetic distances (Kosambi cM) are depicted at the right and left sides of chromosome 9, respectively. (B). LOD value profile from MQM mapping of α-tocopherol concentration on chromosome 9. *Y*-axis corresponds to the genetic map with distances expressed in (A). Horizontal line corresponds to the LOD value. Solid red and dashed blue lines indicate the LOD scores calculated using F_2 _seed and F_2 _plant population, respectively.

For α-tocopherol concentration, only one QTL was detected. The QTL was located on a linkage group K (chromosome 9). MQM analysis revealed that an interval between Sat_243 and KSC138-17 had a strong correlation with α-tocopherol concentration, with LOD value 23.4 and phenotypic variation explained (PVE) by this QTL of 55.8% (Figure 2, Table 1). In our previous study [18], there was a strong correlation between α-tocopherol concentration and γ-tocopherol concentration. Therefore, the QTL analysis was conducted not only for α-tocopherol but also for γ-tocopherol and δ-tocopherol. This was done to elucidate the relationship among tocopherol isoforms and to identify the gene(s) that determine tocopherol composition. From MQM mapping, the QTL located in an interval between Sat_243 and KSC138-17 was also associated with γ-tocopherol concentration (LOD = 11.5, PVE = 32.8%) and δ-tocopherol concentration (LOD = 5.0, PVE = 16.1%).

**Table 1:** QTL associated with tocopherol concentration or content using F_2 _seed and F_2 _plant populations.

Population	Trait^a^	LOD^b^	PVE (%)^c^	Add ^d^
F_2 _seed	α%	23.4	55.8	4.158
	γ%	11.5	32.8	-2.585
	δ%	5.0	16.1	-1.553

F_2 _plant	α%	20.2	55.0	8.009
	γ%	16.7	48.7	-6.163
	δ%	4.8	17.0	-1.836
	α-content	20.6	56.5	1.160
	γ-content	5.24	17.9	-1.094

QTLs are detected using multiple QTL mapping (MQM) method in MapQTL 5. Permutation test (1000 times) was performed to determine genome wide significance threshold level (*P *< 0.05).

^a^α% represents α-tocopherol concentration, γ% represents γ-tocopherol concentration, δ% represents δ-tocopherol concentration, α-content represents α-tocopherol content (μg per 100 mg dry weight seeds), and γ-content represents γ-tocopherol content (μg per 100 mg dry weigh seeds). ^b^LOD means logarithm of odds, the peak of LOD value in the QTL range. ^c^PVE means the percentage of phenotypic variance explained for the trait. ^d^Positive values of additive effect (Add) mean the increased effect for the QTL was caused by KAS allele.

For the F_2 _plant population, QTLs for tocopherol concentrations and contents were analyzed. The same QTL observed in the analysis of the F_2 _seed population was also detected for α-tocopherol concentration (LOD = 20.2, PVE = 55.0%), γ-tocopherol concentration (LOD = 16.7, PVE = 48.7%), and δ-tocopherol concentration (LOD = 4.8, PVE = 17.0%). Moreover, this QTL was also responsible for α-tocopherol content (LOD = 20.6, PVE = 56.5%) and γ-tocopherol content (LOD = 5.24, PVE = 17.9%). For δ-tocopherol concentration, another QTL was detected in interval Sat_244 and Sat_033 of linkage group M (chromosome 12), with LOD value 5.26 and PVE 22.5%. However, this QTL was not detected in F_2 _seeds analysis.

It has been reported that four QTLs for tocopherol concentrations and contents were detected from QTL analysis in a segregating population derived from a cross between a Chinese variety (Hefeng 25) and a high α-tocopherol Canadian variety (OAC Bayfield) [17]. However, in this study, no QTL was detected in those regions. This fact suggests that the genetic factor responsible for high α-tocopherol concentration in KAS may be different from that in OAC Bayfield.

### Identification of candidate gene in the QTL region

**Figure 3:**
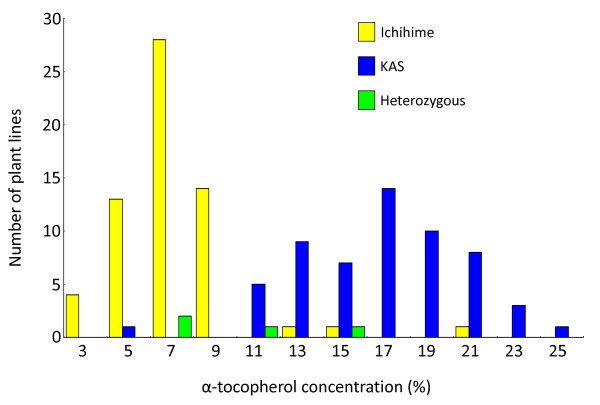
**Frequency distribution of phenotypes and genotypes of marker closely linked for α-tocopherol concentration in F_5 _plant lines**. Frequency distribution of α-tocopherol concentration and genotypes of the KSC138-17 marker in F_5 _plant lines. Yellow, blue, and green bars represent plant lines with Ichihime, KAS, and heterozygous genotypes, respectively.

**Figure 4:**
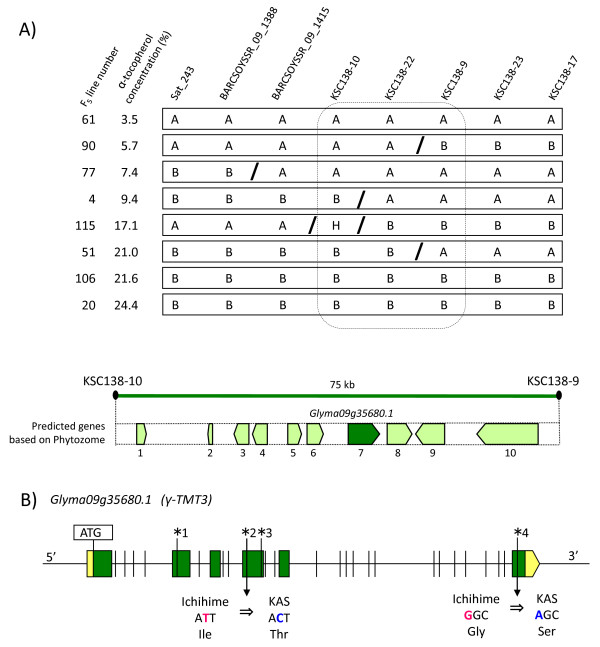
**Graphical genotypes of recombinant plants selected from fine mapping and gene structure of *γ-TMT3***. (A). Summary of informative F_5 _plant lines used for fine mapping of the QTL responsible for high α-tocopherol concentration. Ichihime homozygous genotypes and KAS homozygous genotypes of each marker are represented by 'A' and 'B', respectively. Heterozygous genotype is represented by 'H'. '/' represent recombination positions. The region contributing to high α-tocopherol concentration is enclosed by a dashed box. KSC138-9 genotypes were only analyzed for these informative lines. The interval between KSC138-10 and KSC138-9 corresponded to a 75-kb sequence region on chromosome 9. Based on information from the Phytozome database, the region contained 10 predicted genes. Arrows referred to the genes and numbers below arrows correspond to the numbers in Table 2. (B). Gene structure of *Glyma09g35680.1 *(*γ-TMT3*). The green rectangles and the spaces between the green rectangles represent exons and introns, respectively. The yellow rectangle represents the 5'-UTR region, while the yellow arrow represents the 3'-UTR region. Vertical lines represent genetic polymorphisms (insertion-deletion, SNPs) between Ichihime and KAS. Nucleotide polymorphisms in the exons are indicated by vertical lines and numbers, which are summarized in Table 3. The polymorphisms numbered 2 and 4 are nonsynonymous nucleotide substitutions; the corresponding amino acid changes (Ichihime to KAS) are indicated below the substitution sites.

To identify the candidate gene on chromosome 9, fine mapping was performed in the QTL region flanked by the Sat_243 and KSC138-17 markers using F_5 _lines. The F_5 _lines were derived from the F_2 _plants using single seed descent method. The frequency distribution of α-tocopherol concentration in F_5 _lines is shown in Figure 3. The α-tocopherol concentration was nearly co-segregated with genotypes of KSC138-17 marker (Figure 3). F_5 _lines showing recombination in the region between Sat_243 and KSC138-17 were genotyped for newly developed SSR markers located between Sat_243 and KSC138-17 (Figure 4A). The fine mapping showed that the candidate gene contributing to high α-tocopherol concentration in KAS was likely located in the region between KSC138-10 and KSC138-9, which corresponded to approximately 75 kb of genomic sequence (Figure 4A).

Based on soybean genome information in the Phytozome database [20], there were 10 predicted genes located in the QTL region between KSC138-10 and KSC138-9 on chromosome 9 (Table 2, Figure 4A). One of them, Glyma09g35680.1, shared 81.8% peptide similarity with γ-TMT encoding gene in Arabidopsis, *VTE4 *[21]. *In silico *analysis further revealed that two additional genes encoding γ-TMT exist in the soybean genome: Glyma12g01680.1 and Glyma12g01690.1. Their predicted polypeptides similarity to *VTE4 *was 81.4% and 68.9%, respectively, and both genes were located in tandem on linkage group H (chromosome 12), separated by 4 kb genomic sequence. Interestingly, two γ-TMT genes located in tandem were known to regulate α-tocopherol biosynthesis in sunflower [13]. However, no QTL for α-tocopherol biosynthesis has been found at linkage group H located in tandem with Glyma12g01680.1 and Glyma12g01690.1 in soybean. According to the genome information of database Phytozome [20], there is no the conserved synteny between the genomic regions surrounding Glyma12g01680.1 and Glyma12g01690.1, and Glyma09g35680.1. However, in this study, we were unable to determine whether these regions were homeologous to each other or not.

**Table 2:** Predicted genes located in QTL region, based on information of Phytozome database.

Number^a^	Glyma number	Predicted function
1	Glyma09g35620.1	auxin responsive protein
2	Glyma09g35630.1	auxin responsive protein
3	Glyma09g35640.1	diphtheria toxin resistance
4	Glyma09g35650.1	no function annotation
5	Glyma09g35660.1	amidophosphoribosylpyrophosphate transferase domain
6	Glyma09g35670.1	amidophosphoribosylpyrophosphate transferase domain
7	Glyma09g35680.1	γ-tocopherol methyltransferase (γ-TMT)
8	Glyma09g35690.1	no function annotation
9	Glyma09g35700.1	no function annotation
10	Glyma09g35710.1	DNA topoisomerase type I

^a^Number corresponds to gene number shown in Figure 4A.

**Figure 5:**
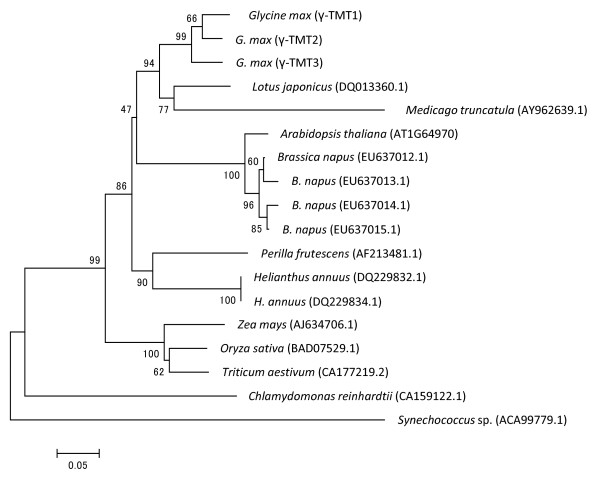
**Neighbor-joining phylogenetic tree of γ-TMT proteins**. Comparison of the deduced amino acid sequences of γ-TMT1, γ-TMT2, and γ-TMT3 from soybean with γ-TMTs of plants, green algae and cyanobacteria. GenBank accession numbers are shown in parentheses. An unrooted tree based on amino acid sequence similarity was obtained by using the neighbor joining method. Bootstrapping was performed with 1,000 replicates, and the bootstrap values (percent) are indicated above the supported branches. The scale bar indicates the distance corresponding to 5 changes per 100 amino acid positions. The predicted protein sequences were initially clustered by using ClustalW.

Glyma12g01680.1 and Glyma12g01690.1 were identical to genomic sequences (*γ-TMT1 *and *γ-TMT2*, respectively) obtained from Ichihime (Ujiie, unpublished data). Therefore, Glyma12g01680.1 and Glyma12g01690.1 were designated as *γ-TMT1 *and *γ-TMT2*, respectively. Glyma09g35680.1 was designated as *γ-TMT3*. Based on predicted amino acid composition, the three γ-TMTs were classified into one phylogenetic group, which is a part of a cluster of γ-TMTs found in dicots (Figure 5).

**Figure 6:**
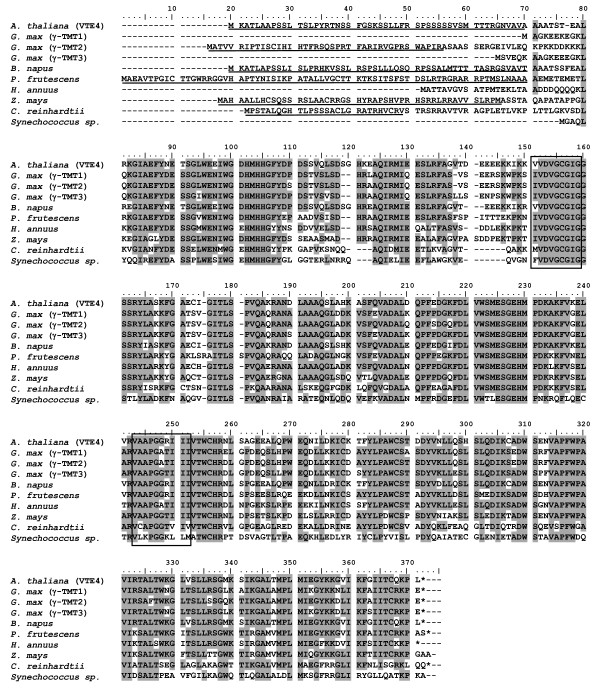
**Amino acid sequence alignment of γ-TMT proteins**. Comparison of the deduced amino acid sequences of γ-TMT1, γ-TMT2, and γ-TMT3 with those of other plants green algae and cyanobacterium. For *B. napus *(EU637012.1) and *H. annuus *(DQ229832.1), only one of the sequences was used for alignment. The sequences were compared with *A. thaliana *γ-TMT (VTE4) as a standard; identical residues in other sequences are shaded, and gaps introduced for alignment purposes are indicated by dashes (-). Lines under amino acid sequences represented plastid transit peptides, which were predicted by using ChloroP1.1 [37]. Blocks surrounded by black boxes are conserved SAM-binding domains, as reported by Shintani and DellaPenna [21].

Except for the N-terminal region, the three γ-TMTs from soybean share high amino acid similarity with γ-TMTs found in several other plant species (Figure 6). The plastid is known as a site for α-tocopherol biosynthesis [11], therefore the existence of plastid transit peptide signals in the three γ-TMT proteins using a prediction program of the subcellular localization was searched. As a result of ChloroP analysis, a plastid transit peptide was predicted in γ-TMT2, but not in γ-TMT1 or γ-TMT3 (Figure 6).

**Figure 7:**
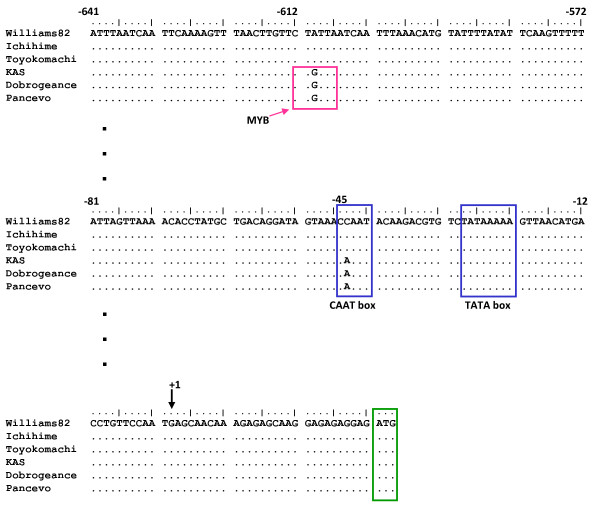
**Predicted transcription factor binding motifs in 5'-upstream sequence of *γ-TMT3***. The 5'-upstream sequence of *γ-TMT3 *was isolated from high α-tocopherol soybeans (KAS, Dobrogeance, and Dobrudza 14 Pancevo [Pancevo]), and typical cultivars (Williams 82, Ichihime, and Toyokomachi). *Cis*-element motifs were predicted by using the PLACE [39] and PLANTCARE databases [40]. Only motifs where nucleotide polymorphisms occur are shown. CAAT: common *cis*-acting and enhancer; MYB: binding site for MYB transcription factor. ATG surrounded by green box indicates translation start site. +1 indicates transcriptional start site (TSS). Numbers above the nucleotides refer to the distance from the TSS. Vertical rows of dots represent promoter regions not shown in the figure.

In this study, QTLs responsible for α-tocopherol concentration and γ-tocopherol concentration were detected at the same location (linkage group K), strongly supporting the negative correlation between α-tocopherol concentration and γ-tocopherol concentration described in the previous report [18]. On the basis of the biosynthetic pathway of tocopherol (Figure 1), γ-TMT plays a pivotal role in determining the relative concentrations of α-tocopherol and γ-tocopherol. Therefore, we focused on characterization of the *γ-TMT3 *gene. According to the Phytozome database, *γ-TMT3 *is 4.3 kb long and consists of six predicted exons. An approximately 5.5 kb genomic region containing the entire sequence of *γ-TMT3 *gene and its 5'-upstream region was sequenced in both Ichihime and KAS. A total of 26 nucleotide polymorphisms were detected in both exons and introns (Figure 4B). Two nucleotide substitutions in the exons led to amino acid alterations. They seemed not to be nucleotide polymorphisms involved in the high α-tocopherol concentration, because Williams 82 which possessed identical nucleotides to KAS at these two positions showed low α-tocopherol concentration same as that of Ichihime (Table 3). Therefore, the 5'-upstream regions from the transcription initiation site of *γ-TMT3 *between high α-tocopherol and typical soybeans were compared. Approximately 1.2 kb of the 5'-upstream region was sequenced in six varieties with high α-tocopherol concentration (KAS, Dobrogeance, and Dobrudza 14 Pancevo) and typical varieties (Ichihime, Toyokomachi, and Williams 82). Sequences alignment revealed that 10 single-nucleotide polymorphisms (SNPs) were observed between the two groups. Of these, two SNPs were located in gene transcriptional regulation domains: a MYB binding site and a CAAT box at positions -612 and -46, respectively, from the predicted transcriptional start site of Williams 82 (Figure 7). The motif of the CAAT box in high α-tocopherol soybeans was "CAAAT", whereas the motif in typical soybeans was "CCAAT". "CCAAT" is the canonical sequence of the CAAT box, but the "CAAAT" motif is also recognized as a CAAT box motif in mammals [22,23]. On the other hand, the MYB binding site ("CTGTTA") was observed only in high α-tocopherol soybeans. The motif is recognized by MYB transcription factors in maize and Arabidopsis [24].

**Table 3:** Polymorphisms in exon region of *γ-TMT3 *gene.

Cultivar name	*1	*2	*3	*4	α-Tocopherol concentration (%)	Harvesting year
Williams 82	T	C	C	A	3.88 ± 0.32	2009
Ichihime	T	T	A	G	1.99 ± 0.08	2008
Toyokomachi	T	C	A	G	4.84 ± 0.58	2008
KAS	G	C	C	A	19.25 ± 2.22	2008
Dobrogeance	G	C	C	A	18.06 ± 2.20	2006
Dobrudza 14 Pancevo	G	C	C	A	19.38 ± 1.14	2008

Ordinary cultivars (Williams 82, Ichihime, and Toyokomachi) and high α-tocopherol cultivars (KAS, Dobrogeance, Dobrudza 14 Pancevo) were used for analysis. Polymorphisms in exons are depicted by *1, *2, *3, *4 (see Figure.4B). α-Tocopherol concentration data are represented as mean ± SD of the values obtained from triplicate experiments. All plants were grown in Hokkaido University experimental farm.

### Relationship between α-tocopherol concentration and expression levels of *γ-TMT *genes

The expression level of *γ-TMT3 *could affect α-tocopherol content and concentration was investigated because the polymorphisms correlated to α-tocopherol concentration were found in the transcriptional regulatory domain of *γ-TMT3*.

F_5_-24, an F_5 _heterogeneous inbred family (HIF) [25] which was heterozygous for the genomic region surrounding *γ-TMT3 *and homozygous throughout almost entire genome was used to generate plants homozygous for the *γ-TMT3 *genomic region from Ichihime and that from KAS; these are referred to as Ichihime lines and KAS lines, respectively. Three lines homozygous for the Ichihime allele (F_5_-24-10, F_5_-24-14, and F_5_-24-15) and three lines homozygous for the KAS allele (F_5_-24-7, F_5_-24-18, and F_5_-24-22) were generated. From each plant, developing seeds were collected at 30, 40, and 50 days after flowering (DAF).

**Figure 8:**
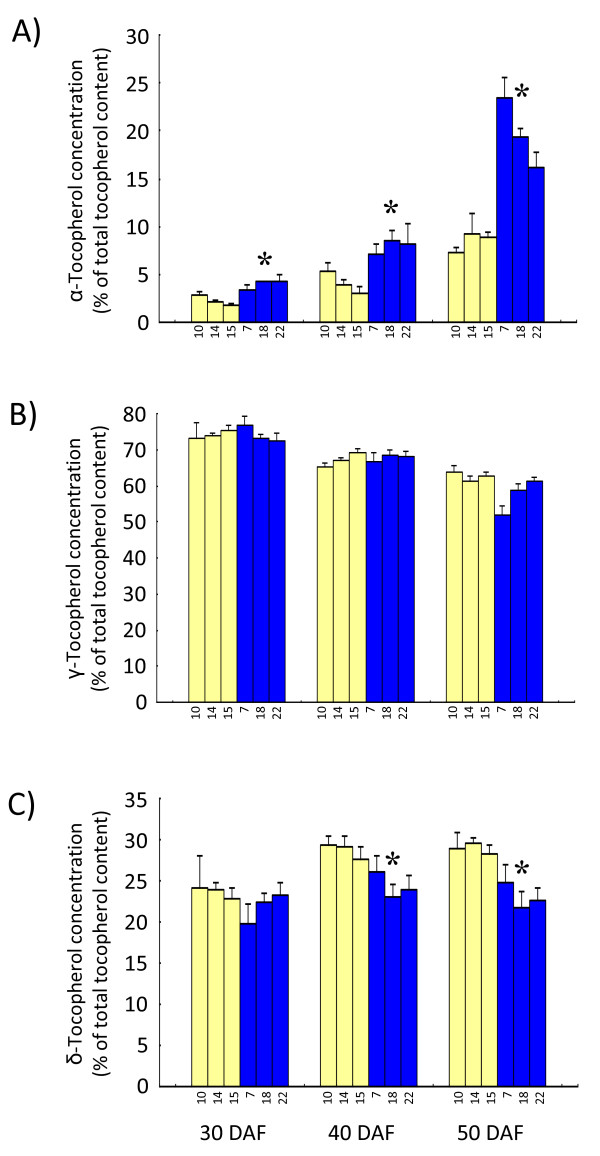
**Tocopherol concentration in developing seeds of HIF-derived lines**. Developing seeds of HIF-derived lines homozygous for either the Ichihime allele for *γ-TMT3 *(F_5_-24-10, F_5_-24-14, and F_5_-24-15; yellow bars) or the KAS allele for *γ-TMT3 *(F_5_-24-7, F_5_-24-18, and F_5_-24-22; blue bars) were used for analysis. Seeds were analyzed at 30 days after flowering (DAF), 40 DAF, and 50 DAF. The concentrations of α-tocopherol (A), *γ*-tocopherol (B), and δ-tocopherol (C) were calculated as the percentage of the tocopherol isoform in total tocopherol content. Data are represented as mean ± SD of the values obtained from triplicate experiments. For each developmental stage, significant differences between the Ichihime genotype group and the KAS genotype group (confidence interval 95%) are shown with asterisks.

As shown in Figure 8A, α-tocopherol concentration increased toward seed maturation. At all developmental stages, the α-tocopherol concentration was significantly higher in the KAS lines than in the Ichihime lines (*P *< 0.05). In 30-DAF seeds, α-tocopherol concentration in the KAS lines was 1.2 to 2.4 times that of the Ichihime lines. The difference between the Ichihime lines and the KAS lines was greater toward seed maturation. At 50 DAF, the α-tocopherol concentration of KAS lines was up to three times that of the Ichihime lines. There was no significant difference (*P *< 0.05) in γ-tocopherol concentration between the Ichihime lines and the KAS lines (Figure 8B). Compared to other tocopherol forms, δ-tocopherol concentration in the KAS lines was significantly lower (*P *< 0.05) than in the Ichihime lines at 40 and 50 DAF (Figure 8C).

**Figure 9:**
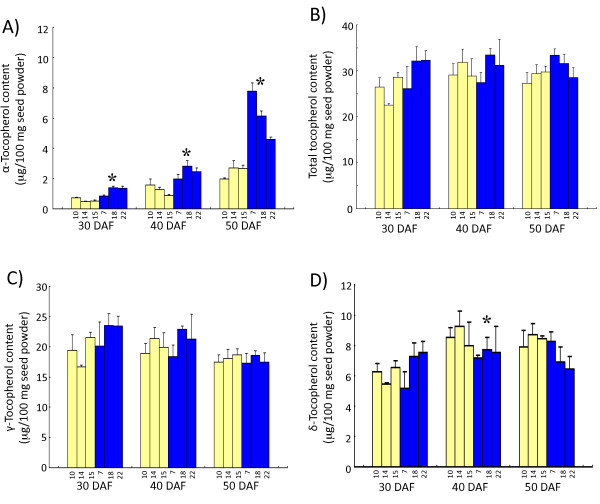
**Tocopherol content in developing seeds of HIF-derived lines**. Developing seeds of HIF-derived lines homozygous for either the Ichihime allele for *γ-TMT3 *(F_5_-24-10, F_5_-24-14, and F_5_-24-15; yellow bars) or the KAS allele for *γ-TMT3 *(F_5_-24-7, F_5_-24-18, and F_5_-24-22; blue bars) were used for analysis. Seeds were analyzed at 30 days after flowering (DAF), 40 DAF, and 50 DAF. The contents of α-tocopherol (A), total tocopherol (B), *γ*-tocopherol (C), and δ-tocopherol (D) were calculated as the weight per 100 milligram dry weight of seed. Data are represented as mean ± SD of the values obtained from triplicate experiments. For each development stage, significant differences between the Ichihime genotype group and the KAS genotype group (confidence interval 95%) are shown with asterisks.

α-Tocopherol content in the KAS lines was significantly higher than that of the Ichihime lines at all seed developmental stages (Figure 9A), and the difference was the greatest at 50 DAF, showing the same tendency as α-tocopherol concentration. In contrast, total tocopherol content did not show significant (*P *< 0.05) change during seed maturation (Figure 9B). It is concluded from these results that the α-tocopherol concentration increase resulted mainly from the increase in α-tocopherol content. Among the other tocopherol forms, γ-tocopherol decreased slightly toward seed maturation, whereas δ-tocopherol content increased until 40 DAF then decreased toward maturation (Figure 9C and 9D). A significant difference (*P *< 0.05) between the KAS lines and the Ichihime lines was observed for δ-tocopherol content at 40 DAF stage, and a slight but not significant difference (*P *< 0.05) between KAS lines and Ichihime lines was also observed for δ-tocopherol content at 50 DAF stage. No significant difference (*P *< 0.05) was observed for γ-tocopherol content at any developmental stage (Figure 9C).

**Figure 10:**
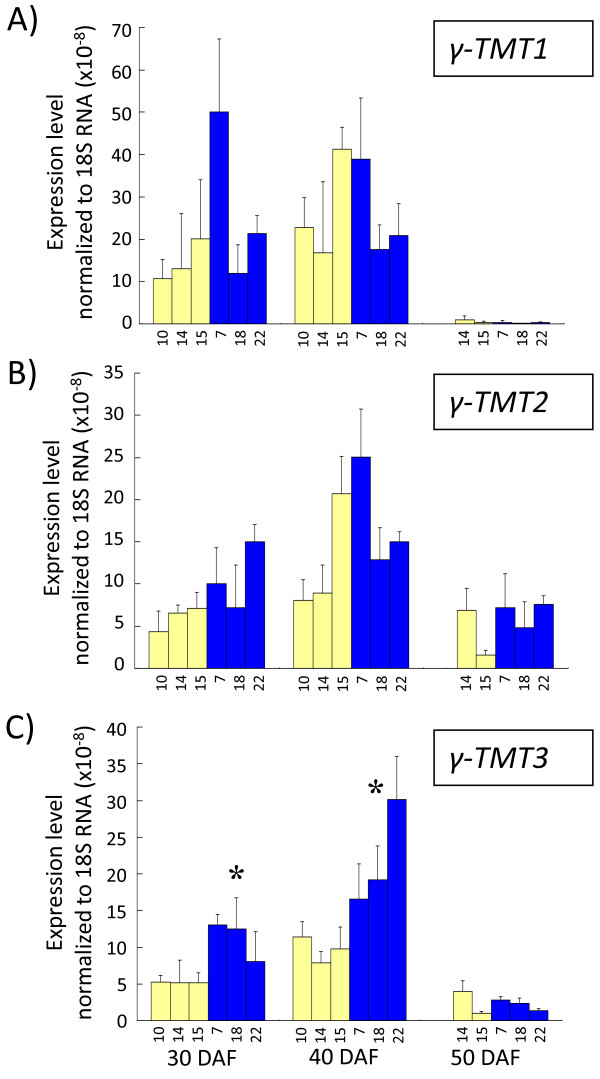
**Gene expression analysis of *γ-TMT1 *(A), *γ-TMT2 *(B), and *γ-TMT3 *(C) during seed development**. Quantitative RT-PCR was performed on total RNA from 30 DAF, 40 DAF, and 50 DAF developing seeds by using the gene-specific primers listed in Table 4. Yellow bars represent HIF-derived homozygous Ichihime lines (F_5_-24-10, F_5_-24-14, and F_5_-24-15) and blue bars represent HIF-derived homozygous KAS lines (F_5_-24-7, F_5_-24-18, and F_5_-24-22). For 30 DAF, and 40 DAF seeds, analysis was performed with all six lines, whereas for 50 DAF seeds, analysis was conducted with five of the six lines (F_5_-24-14, F_5_-24-15, F_5_-24-7, F_5_-24-18, and F_5_-24-22). Transcript levels were normalized with the values obtained for the internal control 18S-ribosomal RNA. Values represent the mean of three replicates ± SD. Asterisks show significant differences between the Ichihime genotype group and the KAS genotype group (confidence interval 95%).

**Figure 11:**
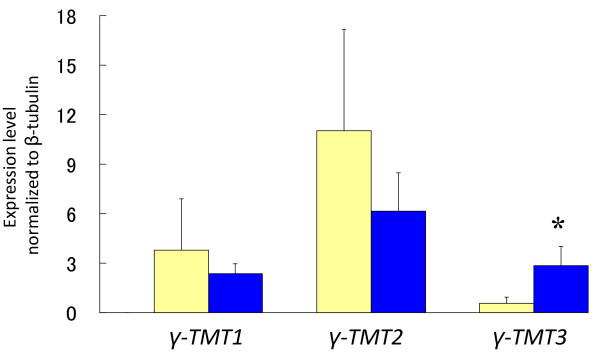
**Gene expression analysis of *γ-TMT1*, *γ-TMT2*, and *γ-TMT3 *in leaves of Ichihime and KAS**. Quantitative RT-PCR was performed on total RNA from leaves using the gene-specific primers listed in Table 5. Yellow bars represent Ichihime and blue bars represent KAS. Transcript levels were normalized with the values obtained for the internal control (β-tubulin mRNA). Values represent the mean of three replicates ± SD. Asterisks show significant difference between the Ichihime genotype group and the KAS genotype group (confidence interval 95%).

The expression levels of *γ-TMT1*, *γ-TMT2 *and *γ-TMT3 *were evaluated by quantitative RT-PCR at three seed developmental stages (Figure 10). The expression level was normalized based on the expression of a reference gene, 18S rRNA which was given as a proper reference gene in a gene expression analysis [26]. The expression of all three *γ-TMT *genes reached the highest level at 40 DAF, when seed size reached the maximum. *γ-TMT1 *and *γ-TMT2 *showed no difference (*P *< 0.05) in expression level between the Ichihime lines and the KAS lines. *γ-TMT3 *showed significant differences (*P *< 0.05) in expression between the Ichihime lines and the KAS lines at both 30 and 40 DAF. The expression level of *γ-TMT3 *in the KAS lines was 1.5 to 3 times that of the Ichihime lines at 30 and 40 DAF (*P *< 0.05). Expression levels of *γ-TMT1*, *γ-TMT2*, and *γ-TMT3 *were also analyzed in fully expanded leaves of Ichihime and KAS. Interestingly, the transcriptional level of *γ-TMT3 *in KAS leaves was also higher than that in Ichihime leaves, the same pattern as was observed in developing seeds (Figure 11).

### Activity of *γ-TMT3 *promoter of Ichihime and KAS

**Figure 12:**
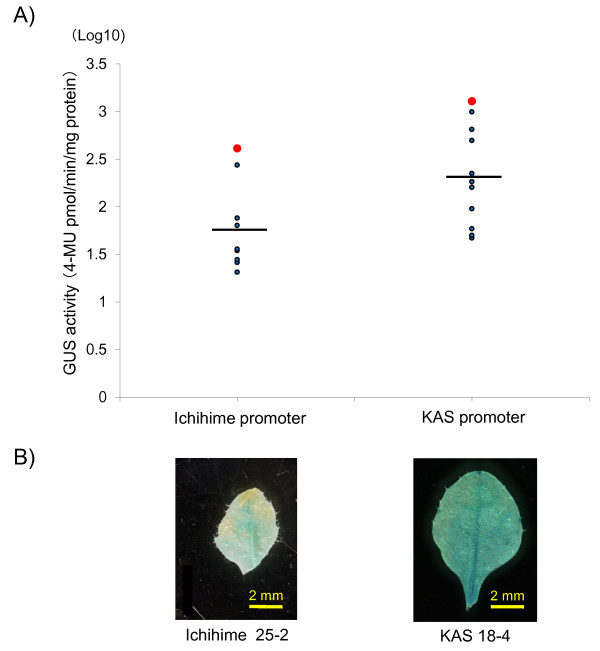
**GUS activity and histochemical analyses of transgenic Arabidopsis plants**. (A). Log-transformed values of GUS activity of T_2 _plants harboring Ichihime or KAS *γ-TMT3 *promoter are shown. Each blue dot represents value of independent T_2 _plant. Red dots represent T_2 _plants with the highest GUS activity, Ichihime 25-2 for Ichihime promoter and KAS 18-4 for KAS promoter. Black bar represents average value of T_2 _plants. (B). GUS histochemical anlaysis of leaves from T_2 _plant harboring Ichihime *γ-TMT3 *promoter (Ichihime 25-2) and T_2 _plant harboring KAS *γ-TMT3 *promoter (KAS 18-4).

Since the expression level of *γ-TMT3 *was different in leaves as well as in developing seeds (Figure 11), we measured the transient activities of *γ-TMT3 *promoters in transgenic Arabidopsis leaves expressing GUS reporter gene under the control of *γ-TMT3 *promoter from KAS or Ichihime. The GUS activity of 10 T_2 _plants carrying the *γ-TMT3 *promoter from Ichihime and 11 T_2 _plants carrying the *γ-TMT3 *promoter from KAS were shown in Figure 12A and 12B. Mean of the GUS activity in transformants carrying *γ-TMT3 *promoter of KAS was 385.5 pmol 4-MU min^-1 ^mg^-1 ^protein, whereas the mean in transformants with Ichihime promoter was 100.53 pmol 4-MU min^-1 ^mg^-1 ^protein. F test analysis for log-transformed data showed that the activity of *γ-TMT3 *promoter of KAS was significantly higher than that of Ichihime promoter (F = 7.170, *P *= 0.015).

## Discussion

### *γ-TMT3 *is the candidate gene for high α-tocopherol concentration in KAS

In the previous study, two SSR markers, Sat_243 and Sat_167 on a linkage group K (chromosome 9) were strongly associated with α-tocopherol concentration. In this study, we confirmed that the QTL in interval Sat_243 and KSC138-17 was associated with α-tocopherol concentration, γ-tocopherol concentration, α-tocopherol content, and γ-tocopherol content. The QTL positively regulated α-tocopherol concentration and α-tocopherol content, and negatively regulated γ-tocopherol concentration and γ-tocopherol content (Table 1), indicating that the candidate gene is directly related to conversion of γ-tocopherol to α-tocopherol. Fine mapping using F_5 _lines showed that *γ-TMT3 *was located in a QTL region. This study focused on the molecular characterization of *γ-TMT3 *gene.

Based on sequencing analysis and gene expression analysis, the nucleotide polymorphisms in *γ-TMT3 *promoter region might increase the expression level of *γ-TMT3 *in developing seeds of KAS, and subsequently associated with high α-tocopherol concentration in KAS seeds. Transient GUS assay for the 1.2-kb promoter region of *γ-TMT3 *from KAS and Ichihime also supported our view that different *γ-TMT3 *expression between KAS and Ichihime could be, at least partly, attributed to the difference in the promoter sequence, although we cannot exclude the possibility that some cis-elements affecting the *γ-TMT3 *expression is located outside of 1.2-kb upstream of the transcriptional start site.

Two of the polymorphisms were located in transcription factor binding motifs in the 5'-upstream region of the *γ-TMT3 *gene in high α-tocopherol soybeans (Figure 7). The first mutation is located in a CAAT box, which acts as an enhancer for gene expression. The canonical sequence of CAAT box is "CCAAT", which is the sequence found in Ichihime. The KAS type is "CAAAT", which is not canonical but is recognized as a functional CAAT box in mouse [22]. At present, we do not know any report that mutation in a CAAT box can enhance gene expression. The second mutation produced a MYB binding site in the KAS promoter; this same sequence ("CTGTTA") is also found in the caffeic acid O-methyltransferase gene promoter of Arabidopsis [24]. In Arabidopsis, the "CTGTTA" motif is recognized by maize MYB transcription factors ZmMYB31 and ZmMYB42 [24]. Further analysis of these *cis*-elements will provide information of whether these polymorphisms contribute to alteration in the promoter activity.

### Regulation of tocopherol content and concentration in soybean

The tocopherol content analysis in this study provides important information about regulation of the tocopherol content and concentration in soybean. In the KAS lines, δ-tocopherol content was lower than in Ichihime lines at 40 DAF. However, the content of γ-tocopherol did not differ between KAS lines and Ichihime lines. Since the peaks from γ-tocopherol and β-tocopherol could not be separated by the analytic method used in this study, it is suggested that increase in β-tocopherol content might mask a decrease in the content of γ-tocopherol. Thus, γ-TMT3 may catalyze both γ-tocopherol and δ-tocopherol conversion to α-tocopherol and β-tocopherol, respectively (Figure 1). The δ-tocopherol decrease and α-tocopherol increase in KAS lines also raises the question of whether γ-TMT3 can also catalyze the methylation of MPBQ to DMPBQ. It is reported that Arabidopsis γ-TMT (VTE4) was not active toward MPBQ *in vitro *[27]. In soybean, there was little similarity in amino acid sequences between γ-TMTs and MPBQ-MTs, indicating that soybean γ-TMTs might not be active toward MPBQ. Further analysis of the enzymatic activity and substrate specificity of γ-TMT3 will provide more information about the biochemical properties of γ-TMT3.

### The possibility of functional differentiation of γ-TMT proteins

γ-TMT1, γ-TMT2, and γ-TMT3 proteins have amino acid similarity more than 90% and two SAM binding domains (Figure 6), suggesting that they all possess the γ-TMT activity that catalyzes the conversion of γ-tocopherol to α-tocopherol. It is elucidated that three *γ-TMT *genes (*γ-TMT1*, *γ-TMT2*, and *γ-TMT3*) were expressed in leaves and developing seeds where α-tocopherol was synthesized and accumulated (Figure 10, Figure 11). However, it is indicated that alteration in expression level of *γ-TMT3 *alone could increase both α-tocopherol concentration and α-tocopherol content to up to 2.4 times that of typical soybean (Figure 8A, 9A). If *γ-TMT1 *or *γ-TMT2 *mutations are also able to enhance α-tocopherol accumulation, gene pyramiding of these *γ-TMT *variants will enable us to develop new soybean varieties with higher α-tocopherol concentration or content than KAS. γ-TMT1, γ-TMT2, and γ-TMT3 polypeptides showed differences in their NH2-terminal region (Figure 6), although they shared high amino acid similarity with γ-TMTs found in several other plant species (Figure 6). Interestingly, no plastid signal peptide was predicted in γ-TMT1 and γ-TMT3 based on *in silico *analysis. α-Tocopherol is known to be localized and be synthesized in plastids [11], and enzymes involved in its biosynthesis are localized inside the plastid [11,28]. Further analysis about the subcellular localization of γ-TMT1 and γ-TMT3 might elucidate the functional diversifications in γ-TMT proteins for the regulation of α-tocopherol biosynthesis in soybean.

## Conclusions

In this work, we identified a QTL responsible for genetic regulation of the high α-tocopherol concentration in KAS. In addition to regulating α-tocopherol concentration, this QTL also affected γ-tocopherol concentration and δ-tocopherol concentration. Thus it is suggested that a gene underlying this QTL regulates tocopherol concentration. Through fine mapping, *γ-TMT3 *was identified as a candidate gene for the high α-tocopherol concentration trait. *γ-TMT3 *encodes γ-tocopherol methyltransferase, which catalyzes the methylation γ-tocopherol to α-tocopherol. The expression of *γ-TMT3 *in the developing seeds of KAS lines was higher than in the seeds of Ichihime lines. Concomitantly, *γ-TMT3 *expression was higher in leaves of KAS than in those of Ichihime. Taken these results together, it is concluded that the promoter region polymorphisms caused higher *γ-TMT3 *expression in KAS, resulting in a higher α-tocopherol concentration. A transient activity analysis of *γ-TMT3 *promoters showed that the activity of KAS *γ-TMT3 *promoter was higher than that of Ichihime *γ-TMT3 *promoter. In this study, it is also demonstrated that genetic variation in the promoter region of *γ-TMT3 *was associated with both α-tocopherol content and concentration in soybean seeds.

## Methods

### Plant material and growing conditions

A total of 140 F_2 _seeds derived from crosses between Ichihime and KAS were used for QTL mapping. The distal portion of each seed was cut off and used for tocopherol concentration analysis. The F_2 _seeds were grown in commercial potting soil (Katakura Chikkarin Co., Ltd., Japan) in the greenhouse of Hokkaido University, Japan (43°0'N, 141°21'E) in 2005. Ten seeds from each plant were collected and bulked for tocopherol concentration analysis. Leaves were harvested from each plant, frozen immediately in liquid nitrogen and stored at -30°C until DNA extraction.

For gene expression and tocopherol quantification analysis in developing seeds, HIF-derived lines were used. An HIF (F_5_-24) was identified as being heterozygous around the *γ-TMT3 *locus based on the genotypes of the SSR markers at flanking loci. The plant was selfed to obtain lines that were homozygous for either Ichihime or KAS marker alleles around the *γ-TMT3 *locus. Three lines homozygous for the Ichihime alleles (24-10, 24-14, 24-15) and three lines homozygous for the KAS alleles (24-7, 24-18, 24-22) were used for analysis; these sets of lines are referred to as Ichihime lines and KAS lines, respectively. All lines were grown at the Hokkaido University experimental farm in June 2008. Seeds of each plant were sampled at 30 days after flowering (DAF), 40 DAF and 50 DAF. The seeds were immediately frozen in liquid nitrogen and stored at -80°C until gene expression and tocopherol content analyses.

### Extraction and HPLC analysis of tocopherols

Tocopherols were extracted from mature seeds and analyzed by reverse-phase high performance liquid chromatography (HPLC) following the procedure described by Dwiyanti *et al*. [18].

For F_2 _seeds, a distal portion of the seed was cut off with razor blade and cut into bits. Ten mg of sample was weighed and sonicated in 300 μl of 80% aqueous ethanol for 10 min at room temperature. Hexane (600 μl) was added to the sample for extraction. The sample was let sit at 4°C before being centrifuged at 13,000 rpm for 5 min using a refrigerated centrifuge (Eppendorf centrifuge 5417R, Eppendorf). The upper (hexane) phase was transferred to an HPLC vial (Waters Corp., Japan). Analysis was performed in an HPLC system (Hitachi LaChrom Elite, Hitachi High-Technologies Corp., Japan) with an Inertsil ODS-3 reverse-phase column (3.0 mm × 250 mm, GL Sciences, Japan). Column temperature was maintained at 40°C and separation was performed under isocratic condition for 25 min. Solvent A was acetonitrile, solvent B was methanol, and the ratio of solvent A to solvent B was 75:25 (v/v). Flow rate was 0.5 ml/min. Tocopherols were detected at the wavelength of 295 nm.

For F_3 _seeds derived from F_2 _plants, five seeds from each plant were bulked and ground into fine powder. Seed powder (50 mg) was weighed into a 15-ml test tube. The powder was sonicated in 1 ml of 80% aqueous ethanol for 15 minutes at room temperature. After incubation at 4°C for 30 min, the sample was centrifuged for 10 min at 2,500 rpm in a Tomy RL-100 centrifuge (Tomy Seiko Co., Japan). The upper phase was transferred to an HPLC vial. Analysis was performed in an HPLC system (Hitachi LaChrom Elite, Hitachi High-Technologies Corp., Japan) with same column as used for the F_2 _seed analysis. Column temperature was maintained at 40°C and separation was performed under isocratic condition for 25 min. The mobile phase was acetonitrile:methanol at a ratio 90:10 (v/v) ratio. Flow rate was 0.5 ml/min. Tocopherols were detected at the wavelength of 295 nm. Each analysis was performed twice.

Tocopherol extraction and quantification of developing seeds were performed based on a procedure developed previously [29] with several modifications. Twenty mg of freeze-dried seed powder was stirred in 1 ml cold acetone. The sample was sonicated at room temperature for 20 min. After the sonication, the sample was incubated at 4°C for 30 min. Centrifugation was performed twice, at 13,000 rpm for 10 min each time using a refrigerated centrifuge (Eppendorf centrifuge 5417R, Eppendorf). The upper solution was transferred into an HPLC vial. The analysis was performed using a Hitachi LaChrom Elite with a reverse-phase column (Inertsil ODS-3, 4.6 mm × 250 mm). The column temperature was maintained at 40°C. The analysis was performed under isocratic condition, with a mobile phase of ethyl acetate:75% methanol at a ratio of 50:50 (v/v). Tocopherols were detected by UV light with the detection wavelength set at 295 nm. Each analysis was performed three times.

Tocopherol content in the sample was calculated against the peak area of dl-tocol (Tama Biochemical Co. Ltd. Japan). dl-Tocol was added into the 80% ethanol or acetone used in the extraction at a concentration of 3 μg/ml.

### Genotyping

Leaves from each F_2 _plants were sampled and stored at -30°C until DNA extraction. Genomic DNA isolation was performed according to the CTAB method as described by Dwiyanti [18]. About 0.2 g of leaf tissue ground in liquid nitrogen was added to 700 μl of cetyl trimethyl ammonium bromide (CTAB) extraction buffer. After 30 min incubation at 60°C, the extract was mixed with 700 μl of chloroform:isoamyl alcohol (24:1 v/v), and centrifuged at 10,000 rpm for 5 min in a refrigerated centrifuge Tomy MR150 (Tomy Seiko Co., Japan). The aqueous solution was transferred to a 1.5-ml tube, and mixed with 500 μl of cold isopropanol for nucleic acid precipitation. Crude nucleic acids were collected by centrifugation at 10,000 rpm for 5 min in a refrigerated centrifuge Tomy MR150 (Tomy Seiko Co., Japan). The nucleic acid pellet was washed with 150 μl of 70% ethanol and the remaining liquid was evaporated. The pellet was then dissolved in TE buffer. RNA was precipitated by lithium chloride as described in [18]. About 20 ng of total DNA was used as the template for PCR analysis.

SSR markers were selected from the soybean consensus linkage map [30] to cover all soybean linkage groups and tested for polymorphism between Ichihime and KAS. Additional SSR markers were developed based on the soybean genomic database Phytozome [20] and soybean SSR database BARCSOYSSR_1.0 [31]. Genotypes of 148 selected SSR markers were determined in F_2 _plants. The DNA band for each marker was amplified by using the PCR procedure described previously [18]. Amplified products were separated on either 3% Agarose S (Wako Pure Chemical Industries, Ltd), 4% NuSieve Agarose S (Cambrex Bio Science Rockland, Inc.), or 10% polyacrylamide gel. The gel was stained with ethidium bromide, and DNA bands were photographed under UV light.

### Genetic mapping and QTL analysis

A linkage map based on the genotypes of 152 SSR markers in 122 F_2 _plants was constructed using MapManager QTX [32]. Map distances were calculated in centiMorgans (cM) by using the Kosambi function.

QTL analyses for α-tocopherol concentration, γ-tocopherol concentration and δ-tocopherol concentration were carried out in both F_2 _seeds and F_2 _plants. For F_2 _plants, QTL analyses for α-tocopherol content and γ-tocopherol content were also performed. Permutation analysis (1,000 times) was performed to determine the genome-wide minimum significant LOD threshold score. Based on the analysis result, QTLs with LOD score exceeding 2.8 were regarded as effective loci. Initial QTL mapping was performed by using the interval mapping (IM) method provided in MapQTL 5.0 [19]. Markers flanking the QTLs were used as cofactors in QTL mapping by using the MQM method in the same program.

### Fine mapping

F_5 _plants were generated from F_2 _plants by using the single-seed-descent method. These F_5 _plants were planted at the Hokkaido University experimental farm, Japan (43°0'N, 141°21'E) in June 2007. Ten seeds from each plant were bulked for tocopherol concentration analysis, and the leaves of each plant were used for DNA genotyping. Tocopherol quantification was performed with the same method used for F_3 _seeds. DNA was extracted from leaves with the CTAB method.

Six SSR markers (Table 4) were developed to identify recombinants in the region containing the QTL. These markers genotypes were determined in F_5 _plants. The PCR reaction mixture was 20 ng DNA, 1 μl of 10× PCR buffer (TaKaRa), 0.25 mM of dNTP mixture (TaKaRa), 0.2 μM forward primer, 0.2 μM reverse primer and 0.5 units of *Taq *DNA polymerase (TaKaRa) in a total volume of 10 μl. PCR reaction was performed as follows: an initial denaturation step at 95°C for 5 min; followed by 35 cycles of 95°C for 30 s, 58°C for 30 s, and 72°C for 30 s; followed by a final extension step at 72°C for 7 min. PCR products were separated on 10% acrylamide gels, and bands were visualized under UV illumination.

**Table 4:** Primers used for fine mapping.

Primer name	Direction	Nucleotide sequence (5' to 3')
KSC138-9	Forward	GCACAATAAATTGGGCCTGA
	Reverse	GCGAGTGTTGGGCTAAGTCT

KSC138-10	Forward	CACGAATGTGAATTTGATCG
	Reverse	CGACCAAGGAGATAAAAACAGA

KSC138-17	Forward	TGGAAATTCTGTGCACTTGGTG
	Reverse	TAAAGCCGCCTAGCCGATTG

KSC138-22	Forward	TGCAGCAATAATCAATCAAATAGAA
	Reverse	TTCAATCAAATTTAGCACGTGTATT

KSC138-23	Forward	CGGTCCAGATTTAATTCTTTCACTC
	Reverse	TTTCCGTTTTGTCACCCTGCT

BARCSOYSSR_09_1388	Forward	TTGCACTCTCCAAACCAAGA
	Reverse	ATGCACTCTGCTCGACACAT

BARCSOYSSR_09_1415	Forward	CACCATCCACTCCAGTTCCT
	Reverse	CTCCACGTGTTAGACGGGTT

### Phylogenetic analysis and plastid transit peptide prediction

Amino acid sequences of γ-TMT1, γ-TMT2, and γ-TMT3 were obtained from the Phytozome database [20]. Amino acid sequences of γ-TMT homologs from cyanobacteria (*Synechococcus *sp. PCC 7002 [ACA99779.1]), green algae (*Chlamydomonas reinhardtii *[CA159122.1]), plants (*Lotus japonicus *[DQ013360.1], *Medicago truncatula *[AY962639.1], Arabidopsis [AT1G64970], sunflower [DQ229832.1 and DQ229834.1], rapeseed [EU637012.1, EU637013.1, EU637014.1. EU637015.1], maize [AJ634706.1], rice (*Oryza sativa *L.) [BAD07529.1], wheat (*Triticum aestivum *L.) [CA177219.2], and *Perilla frutescens *[AF213481.1]) were obtained from TAIR [33] and NCBI GenBank [34]. The sequences were aligned by the ClustalW function in MEGA 4.0 software [35]. A phylogenetic tree of the proteins was constructed by using the neighbor-joining method in MEGA 4.0 software [35]. A bootstrap (resampling) test was performed 1,000 times to determine the distances between proteins. Plastid transit peptide prediction was performed using ChloroP 1.1 [36].

### Gene cloning and sequencing

Genomic DNA samples from high α-tocopherol soybean varieties (KAS, Dobrogeance, and Dobrudza 14 Pancevo) and typical soybean varieties (Ichihime, Toyokomachi, and Williams 82) were isolated by the CTAB method described in the genotyping section. Primer pairs were designed based on *γ-TMT3 *(Glyma09g35680.1) genomic information [20]. *γ-TMT3 *fragments were amplified by using the following PCR conditions: initial denaturation step at 95°C for 5 min; followed by 35 cycles of 95°C for 30 s, annealing temperature for 30 s, 72°C for 1 min; followed by a final extension step at 72°C for 7 min. PCR products were separated in 1% Agarose S gel (Wako Pure Chemical Industries, Ltd). Expected amplification products were excised from the gel, precipitated with ethanol and ligated into the pGEM-T Easy vector (Promega Corp.). Vectors containing DNA fragments were transformed into *Escherichia coli *strain JM109. After overnight culture, plasmids were isolated by using Wizard SV Plus Minipreps (Promega Corp.). DNA fragments were treated with a Big Dye Terminator Cycle Sequencing ver.3.1 kit (Applied Biosystems) with the following reaction conditions: 30 cycles of 96°C for 10 s, 50°C for 5 s and 60°C for 2 min. DNA fragments were sequenced by using an ABI PRISM 3130 Genetic Analyzer (Applied Biosystems) and the sequences were aligned using the BioEdit Sequence Alignment Editor [37].

### RNA extraction

Total RNA was extracted from developing seeds or leaves following the lithium chloride precipitation procedure [38] with several modifications. After frozen tissue (about 200 mg) was ground to a fine powder in liquid nitrogen, 150 μl of Tris-saturated phenol (pH 8.0) and 500 μl of extraction buffer (10 mM Tris-HCl pH 7.5, 1 mM EDTA pH 8.0, 100 mM NaCl, 1% SDS) were added to the frozen powder. The mixture was ground thoroughly. Three-hundred μl of chloroform:isoamyl alcohol (24:1 v/v) was added to the sample, the solution was vortexed, and the aqueous and organic layers were separated by centrifugation (15,000 rpm, 10 min, 4°C) in a refrigerated centrifuge (HITACHI Himac CF15RX II, Tokyo, Japan). The aqueous phase was transferred into a 1.5-ml tube. The chloroform:isoamyl alcohol treatment was performed twice. The RNA was precipitated by the addition of 0.3 volumes of 10 M lithium chloride. After being stored at 4°C overnight, the solution was centrifuged (15,000 rpm, 15 min, 4°C). The RNA pellet was dried by leaving the tube opened on ice. The RNA pellet was resuspended in RNase free water.

DNA was removed from the resuspended pellet by DNase I treatment. Ten units of DNase I (TaKaRa) and DNase I buffer was added into the RNA solution. The mixture was incubated at 37°C for 30 min. RNA was precipitated again in the presence of 0.3 M sodium acetate and 2.5 volumes of ethanol. The RNA pellet was dried, and again resuspended in RNase free water.

### Quantitative RT-PCR analysis

Each cDNA was synthesized from 1 μg of total RNA by using the M-MLV reverse transcriptase system (Invitrogen) with random hexamer primers according to the manufacturer's instructions. After synthesis, one volume of cDNA was diluted with four volumes of nuclease-free water.

The quantitative RT-PCR reaction was conducted in a 20-μl volume containing 5 μl of cDNA, 12.5 mol of each primer and 2× SYBR Premix Ex Taq II (Applied Biosystems). The reaction was performed in a DNA Engine Opticon3 (MJ Research Inc.) under the following conditions: 40 cycles of 95°C for 20 s, 58°C for 20 s and 72°C for 20 s. The specificity of the amplification was verified by melting-curve analysis. The expression levels of the *γ-TMT *genes were normalized to the level of 18 rRNA for developing seeds analysis, and to β-tubulin for leaf analysis. Primers used for each *γ-TMT *gene, 18S rRNA and β-tubulin are summarized in Table 5.

**Table 5:** Primers used for gene expression analysis.

Primer name	Direction	Nucleotide sequence (5' to 3')
γ-TMT1	Forward	CTGGAGGCAGAGTATAGCG
	Reverse	AAACTCCCAGGTCCCACCCAAT

γ-TMT2	Forward	GAAGCAAGTTTCCAACAGGTCG
	Reverse	CGCCAATCATAGGAGATATTGCATATG

γ-TMT3	Forward	CAGTGGACTTAAAACCATAAAGGGAGC
	Reverse	CCACATACTCTATATCATTCACACGAG

18S rRNA	Forward	TGATTAACAGGGACAGTCGG
	Reverse	ACGGTATCTGATCGTCTTCG

β-tubulin	Forward	GAGAAGAGTATCCGGATAGG
	Reverse	GAGCTTGAGTGTTCGGAAAC

### Bioinformatic analysis of the promoter sequences

The upstream 1.3 kb regions of *γ-TMT3 *from Ichihime, Toyokomachi, Williams 82, KAS, Dobrogeance, and Dobrudza 14 Pancevo were analyzed. Regulatory elements in these regions were analyzed using program PLACE [39] and PLANTCARE [40].

### Generation of transgenic Arabidopsis harboring GUS gene under the control of *γ-TMT3 *promoter

The 1.2 kb region upstream the transcriptional start site in the *γ-TMT3 *promoter was amplified from Ichihime and KAS, cloned into PCR^®^8/GW/TOPO^® ^vector (Invitrogen). The plasmids were sequenced. The promoter fragments were inserted into a plant expression vector pMDC100 [41] containing a β-glucuronidase (GUS) reporter gene [42]. The construct was introduced into *Agrobacterium tumefaciens *strain EHA105. *Arabidopsis thaliana *ecotype Columbia plants were transformed with *A. tumefaciens *harboring the expression vector using a floral-dip method [43].

### GUS histochemical and activity analyses

For GUS histochemical assay of transgenic Arabidopsis, leaves from T_2 _plants were soaked with staining solution containing 1 mg ml^-1 ^of 5-bromo-4-chloro-3-indoyl-β-d-glucuronide (X-Gluc) based on protocol described by [44]. The soaked leaves were vacuumed for 10 minutes and incubated overnight at 37°C. The chlorophylls were removed by a rinse with 99.5% ethanol after staining treatment.

For GUS activity assay, crude protein was extracted from leaves of T_2 _plants with 200 μl of extraction buffer containing 50 mM of sodium phosphate (pH 7.0), 10 mM of EDTA (pH 8.0), 0.1% of SDS, and 0.1% of Triton X-100. Sixteen μl of the extract was mixed with 50 μl of 1 mM 4-methylumbelliferyl-β-D-glucuronide (4-MUG) and 34 μl of extraction buffer, and incubated at 37°C for 0 min, 30 min, and 60 min. The reactions were stopped by adding 200 μl of 0.2 M sodium carbonate. The fluorescence of 4-methylumbelliferone (4-MU) derived from the reaction was measured using Wallac ArvoTM 1420 Multilabel Counter (Perkin Elmer). Protein content in the extracts was determined using Quick Start™ Bradford Protein Assay Kit (Bio-Rad Laboratories). GUS activity was expressed as pmol 4-MU·min^-1^·mg protein.

### Accession Numbers

Sequence data from this article can be found in the GenBank/EMBL/DDBJ data libraries under the following accession number: Ichihime *γ-TMT3 *promoter (AB617792), KAS *γ-TMT3 *promoter (AB617793), Toyokomachi *γ-TMT3 *promoter (AB617794), Williams 82 *γ-TMT3 *promoter (AB617799), KAS *γ-TMT3 *coding sequence (AB617795), Ichihime *γ-TMT3 *coding sequence (AB617796), KAS *γ-TMT3 *genome (AB617797), and Ichihime *γ-TMT3 *genome (AB617798).

## Competing interests

The authors declare that they have no competing interests.

## Authors' contributions

MSD participated in the conception, design, and performance of all experiments. TY was responsible for the fine mapping and gene expression analysis. MS was involved in the analysis for genetic polymorphism. JA was involved in the genetic analysis of the mapping population. KK was responsible for the evaluation of seed contents and participated in experimental conception. All authors contributed to writing of the manuscript. All authors read and approved the final manuscript.

## References

[B1] Bramley PM Elmadfa I Kafatos A Kelly FJ Manios Y Roxborough HE Schuch W Sheehy PJA Wagner K-H Vitamin E J Sci Food Agric 2000 80 913 938 10.1002/(SICI)1097-0010(20000515)80:7<913::AID-JSFA600>3.0.CO;2-3

[B2] Herbers K Vitamin production in transgenic plants J Plant Physiol 2003 160 821 829 10.1078/0176-1617-01024 12940549

[B3] Subramaniam S Slater S Karberg K Chen R Valentin HE Wong Y-H Nucleic acid sequences to proteins involved in tocopherol synthesis International patent application WO 01/79472 2001

[B4] Clemente TE Cahoon EB Soybean oil: genetic approaches for modification of functionality and total content Plant Physiol 2009 151 1030 1040 10.1104/pp.109.146282 19783644 PMC2773065

[B5] Hoppe PP Krennrich G Bioavailability and potency of natural-source and all-racemic alpha-tocopherol in the human: a dispute Eur J Nutr 2000 39 183 193 10.1007/s003940070010 11131364

[B6] Van Eenennaam AL Lincoln K Durrett TP Valentin HE Shewmaker CK Thorne GM Jiang J Baszis SR Levering CK Aasen ED Hao M Stein JC Norris SR Last RL Engineering vitamin E content: from Arabidopsis mutant to soy oil Plant Cell 2003 15 3007 3019 10.1105/tpc.015875 14630966 PMC282849

[B7] Ujiie A Yamada T Fujimoto K Endo Y Kitamura K Identification of soybean varieties with high α-tocopherol content Breed Sci 2005 55 123 125 10.1270/jsbbs.55.123

[B8] Tavva VS Kim YH Kagan IA Dinkins RD Kim KH Collins GB Increased α-tocopherol content in soybean seed overexpressing the *Perilla frutescens *γ-tocopherol methyltransferase gene Plant Cell Rep 2007 26 61 70 16909228 10.1007/s00299-006-0218-2

[B9] Christen S Woodall AA Shigenaga MK Southwell-Keely PT Duncan MW Ames BN γ-Tocopherol traps mutagenic electrophiles such as NOx and complements α-tocopherol: Physiological implications Proc Natl Acad Sci USA 1997 94 3217 3222 10.1073/pnas.94.7.3217 9096373 PMC20349

[B10] Jiang Q Christen S Shigenaga MK Ames BN gamma-Tocopherol, the major form of vitamin E in the US diet, deserves more attention Am J Clin Nutr 2001 74 714 722 11722951 10.1093/ajcn/74.6.714

[B11] Munné-Bosch S Alegre L The function of tocopherols and tocotrienols in plants CRC Crc Cr Rev Plant Sci 2002 21 31 57

[B12] Velasco L Perez-Vich B Fernandez-Martinez JM Identification and genetic characterization of a safflower mutant with a modified tocopherol profile Plant Breeding 2005 124 459 463 10.1111/j.1439-0523.2005.01150.x

[B13] Hass CG Tang S Leonard S Traber MG Miller JF Knapp SJ Three non-allelic epistatically interacting methyltransferase mutations produce novel tocopherol (vitamin E) profiles in sunflower Theor Appl Genet 2006 113 767 782 10.1007/s00122-006-0320-4 16896719

[B14] Marwede V Gul MK Becker HC Ecke W Mapping of QTL controlling tocopherol content in winter oilseed rape Plant Breeding 2005 124 20 26 10.1111/j.1439-0523.2004.01050.x

[B15] Rocheford TR Wong JC Egesel CO Lambert RJ Enhancement of vitamin E levels in corn J Am Coll Nutr 2002 21 191S 198S 12071304 10.1080/07315724.2002.10719265

[B16] Gilliland LU Magallanes-Lundback M Hemming C Supplee A Koornneef M Bentsink L DellaPenna D Genetic basis for natural variation in seed vitamin E levels in *Arabidopsis thaliana* Proc Natl Acad Sci USA 2006 103 18834 18841 10.1073/pnas.0606221103 17077148 PMC1693748

[B17] Li H Liu H Han Y Wu X Teng W Liu G Li W Identification of QTL underlying vitamin E contents in soybean seed among multiple environments Theor Appl Genet 2010 120 1405 1413 10.1007/s00122-010-1264-2 20069414 PMC2854347

[B18] Dwiyanti MS Ujiie A Thuy LTB Yamada T Kitamura K Genetic analysis of high α-tocopherol content in soybean seeds Breed Sci 2007 57 23 28 10.1270/jsbbs.57.23

[B19] Van Ooijen JW Kyazma BV MapQTL^® ^5, Software for the mapping of quantitative trait loci in experimental populations 2004 Wageningen, The Netherlands

[B20] Phytozome http://www.phytozome.net/soybean.php

[B21] Shintani D DellaPenna D Elevating the vitamin E content of plants through metabolic engineering Science 1998 282 2098 2100 9851934 10.1126/science.282.5396.2098

[B22] Sakata-Takatani K Matsuo N Sumiyoshi H Tsuda T Yoshioka H Identification of a functional CBF-binding CCAAT-like motif in the core promoter of the mouse pro-alpha 1(V) collagen gene (Col5a1) Matrix Biol 2004 23 87 99 10.1016/j.matbio.2004.03.003 15246108

[B23] Himes JL Xanthopoulos KG Biological Role of the CCAAT/Enhancer-binding Protein Family of Transcription Factors J Biol Chem 1998 273 28545 28548 10.1074/jbc.273.44.28545 9786841

[B24] Fornalé S Sonbol FM Maes T Capellades M Puigdomènech P Rigau J Caparrós-Ruiz D Down-regulation of the maize and Arabidopsis thaliana caffeic acid *O*-methyl-transferase genes by two new maize R2R3-MYB transcription factors Plant Mol Biol 2006 62 809 823 10.1007/s11103-006-9058-2 16941210

[B25] Tuinstra MR Ejeta G Goldsbrough Heterogeneous inbred family (HIF) analysis: a method for developing near-isogenic lines that differ at quantitative trait loci Theor Appl Genet 1997 95 1005 1011 10.1007/s001220050654

[B26] Liebault M Thibivilliers S Bilgin DD Radwan O Benitez M Clough SJ Stacey G Identification of four soybean reference genes for gene expression normalization The Plant Genome 2008 1 44 54 10.3835/plantgenome2008.02.0091

[B27] Cheng Z Sattler S Maeda H Sakuragi Y Bryant DA DellaPenna D Highly divergent methyltransferases catalyze a conserved reaction in tocopherol and plastoquinone synthesis in cyanobacteria and photosynthetic eukaryotes Plant Cell 2003 15 2343 2356 10.1105/tpc.013656 14508009 PMC197300

[B28] Soll J Schultz G Joyard J Douce R Block MA Localization and synthesis of prenylquinones in isolated outer and inner envelope membranes from spinach chloroplasts Arch Biochem Biophys 1985 238 290 299 10.1016/0003-9861(85)90167-5 3985624

[B29] Wang S Kanamaru K Li W Abe J Yamada T Kitamura K Simultaneous accumulation of high contents of α-tocopherol and lutein is possible in seeds of soybean (G*lycine max *(L.) Merr.) Breed Sci 2007 57 297 304 10.1270/jsbbs.57.297

[B30] Song QJ Marek LF Shoemaker RC Lark KG Concibido VC Delannay X Specht JE Cregan PB A new integrated genetic linkage map of the soybean Theor Appl Genet 2004 109 122 128 10.1007/s00122-004-1602-3 14991109

[B31] Song QJ Jia GF Zhu YL Grant D Nelson RT Hwang EY Hyten DL Cregan PB Abundance of SSR Motifs and Development of Candidate Polymorphic SSR Markers (BARCSOYSSR_1.0) in Soybean Crop Sci 2010 50 1950 1960 10.2135/cropsci2009.10.0607

[B32] Manly KF Cudmore RH Jr Meer JM Map Manager QTX, cross-platform software for genetic mapping Mamm Genome 2001 12 930 932 10.1007/s00335-001-1016-3 11707780

[B33] The Arabidopsis Information Resource (TAIR) http://www.arabidopsis.org/index.jsp

[B34] National Center for Biotechnology Information http://www.ncbi.nih.gov

[B35] Tamura K Dudley J Nei M Kumar S MEGA4: Molecular Evolutionary Genetics Analysis (MEGA) software version 4.0 Mol Biol Evol 2007 24 1596 1599 10.1093/molbev/msm092 17488738

[B36] Emanuelsson O Nielsen H von Heijne G ChloroP, a neural network-based method for predicting chloroplast transit peptides and their cleavage sites Protein Sci 1999 8 978 984 10.1110/ps.8.5.978 10338008 PMC2144330

[B37] Hall TA BioEdit: a user-friendly biological sequence alignment editor and analysis program for Windows 95/98/NT Nucleic Acids Symp Ser 1999 41 95 98

[B38] Napoli C Lemieux C Jorgensen R Introduction of a chimeric chalcone synthase gene into petunia results in reversible co-suppression of homologous genes in trans The Plant Cell 1990 2 279 289 12354959 10.1105/tpc.2.4.279 PMC159885

[B39] PLACE http://www.dna.affrc.go.jp/PLACE/

[B40] PLANTCARE http://bioinformatics.psb.ugent.be/webtools/plantcare/html/

[B41] Curtis MD Grossniklaus U A gateway cloning vector set for high-throughput functional analysis of genes in planta Plant Physiol 2003 133 462 469 10.1104/pp.103.027979 14555774 PMC523872

[B42] Ohta S Mita S Hattori T Nakamura K Construction and expression in tobacco of a β-glucuronidase (GUS) reporter gene containing an intron within the coding sequence Plant Cell Physiol 1990 31 805 813

[B43] Clough SJ Bent AF Floral dip: a simplified method for Agrobacterium-mediated transformation of Arabidopsis thaliana Plant J 1998 16 735 743 10.1046/j.1365-313x.1998.00343.x 10069079

[B44] Jefferson RA Kavanagh TA Bevan MW GUS fusions: beta-glucuronidase as a sensitive and versatile gene fusion marker in higher plants EMBO J 1987 6 3901 3907 3327686 10.1002/j.1460-2075.1987.tb02730.x PMC553867

